# Donepezil Research in Cognitive Impairment: A Bibliometric and Scientometric Analysis of Global Trends and Pharmacological Perspectives

**DOI:** 10.1002/brb3.71251

**Published:** 2026-02-10

**Authors:** Wencai Wang, Yinuo Chen, Zijie Xiong, Zun Wang, Wei Ye, Xianfeng Li

**Affiliations:** ^1^ Department of Neurosurgery The Second Affiliated Hospital of Harbin Medical University Harbin China

**Keywords:** bibliometric analysis, cognitive impairment, donepezil, knowledge graph analyses, Scientometric Analysis, Web of Science Core Collection

## Abstract

**Background:**

Cognitive impairment (CI) greatly affects global health and quality of life. Donepezil, a widely used treatment for CI, particularly in Alzheimer's disease, has been extensively studied; however, a comprehensive bibliometric analysis summarizing global research trends remains limited.

**Methods:**

Relevant English‐language articles and reviews published between 2000 and 2025 were retrieved from the Web of Science Core Collection. CiteSpace and VOSviewer were employed to analyze publication trends, collaborative networks, journal distribution, co‐citation patterns, and keyword co‐occurrence.

**Results:**

A total of 1907 publications were identified. The United States led in both output and citation impact, with the University of Toronto emerging as the most influential institution. The U.S. Department of Health and Human Services provided the greatest funding support. The *Journal of Alzheimer's Disease* was the primary publishing outlet, and Etsuro Mori was the most prolific and influential author. Keyword analysis revealed “Donepezil,” “Alzheimer's disease,” and “Mild cognitive impairment” as dominant terms. Recent hotspots—such as “acetylcholinesterase,” “oxidative stress,” “neuroinflammation,” “tau protein,” and “mechanism”—reflect a shift toward mechanistic and preclinical research.

**Conclusion:**

Research on donepezil for CI has shown consistent growth, evolving from clinical application toward mechanistic exploration and disease modification. Future studies are expected to focus on individualized therapy, combination strategies, and underexplored CI subtypes, aiming to enhance the therapeutic potential and clinical value of donepezil.

## Graphical abstract

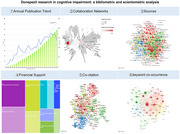

**Abbreviations**
AD: Alzheimer's disease
CI: cognitive impairment
DLB: dementia with Lewy bodies
MCI: mild cognitive impairment
PD: Parkinson's disease
PDD: Parkinson's disease dementia
VaD: vascular dementia
WoSCC: Web of Science Core Collection

## 1 Introduction

Cognitive impairment (CI) encompasses a spectrum of age‐related disorders, extending from mild cognitive impairment (MCI) to severe forms of dementia, including Alzheimer's disease (AD) and Parkinson's disease (PD) (Wu et al. 2017). Affected individuals typically exhibit declines in cognition, including thinking, learning, and memory. With the rapid aging of the global population, CI has become a major public health challenge, imposing substantial health and economic burdens worldwide (Pérez Palmer et al. 2022). The prevalence of MCI among older adults is estimated to range between 6.7% and 25.2% (Jongsiriyanyong and Limpawattana 2018). The prevalence of dementia is estimated to be approximately 5%–7%, with a higher rate observed in women than in men. Furthermore, the prevalence of dementia in individuals aged 100 years and older is 244 times higher than that in those aged 50–59 years (Cao et al. 2020; Lopez and Kuller 2019). Current mainstream management strategies primarily target underlying etiologies (Eshkoor et al. 2015) and combine pharmacological interventions, such as cholinesterase inhibitors (Russ and Morling 2012), with comprehensive approaches involving cognitive training (J. Y. Wang et al. 2024), behavioral modifications, and psychological support.

Donepezil, an acetylcholinesterase inhibitor, has been widely applied in the treatment of various forms of CI (Diaz‐Galvan et al. 2023; Baik et al. 2021; Battle et al. 2021). A randomized controlled trial demonstrated that donepezil treatment improved dual‐task gait speed and dual‐task cost in older adults with MCI (Montero‐Odasso et al. 2019). An experimental study indicated that donepezil may enhance brain‐derived neurotrophic factor expression by inhibiting histone deacetylase 6 (HDAC6) nuclear translocation, thereby alleviating vascular dementia (VaD) in a rat model (Jian et al. 2020). In addition, donepezil is frequently administered in combination with memantine (Padovani et al. 2023) or with a chromone–melatonin hybrid (Pachón‐Angona et al. 2019) for the treatment of AD. These findings highlight the promising application prospects of donepezil in the management of CI and underscore its potential for multidimensional therapeutic benefits.

In recent years, donepezil has been extensively investigated, and substantial progress has been made in its application to the treatment of CI. Nevertheless, this research area has not yet been systematically organized or comprehensively analyzed. Bibliometric analysis provides a systematic approach to organizing relevant literature and quantitatively assessing keywords, citations, authors, countries/regions, and institutions to identify research hotspots and trends (W. Wang et al. 2025). This study applies bibliometric methods to quantitatively analyze research institutions, authors, countries, keywords, and citations within the field of donepezil treatment for CI. It dynamically maps the research frontiers, offering a comprehensive, objective, and systematic data analysis perspective. By addressing gaps in existing literature reviews, this study fulfills the need for researchers to gain a thorough understanding of research trends, key topics, and emerging frontiers in the donepezil research domain.

## 2 Methods

### 2.1 Data Sources and Search Strategy

**FIGURE 1:**
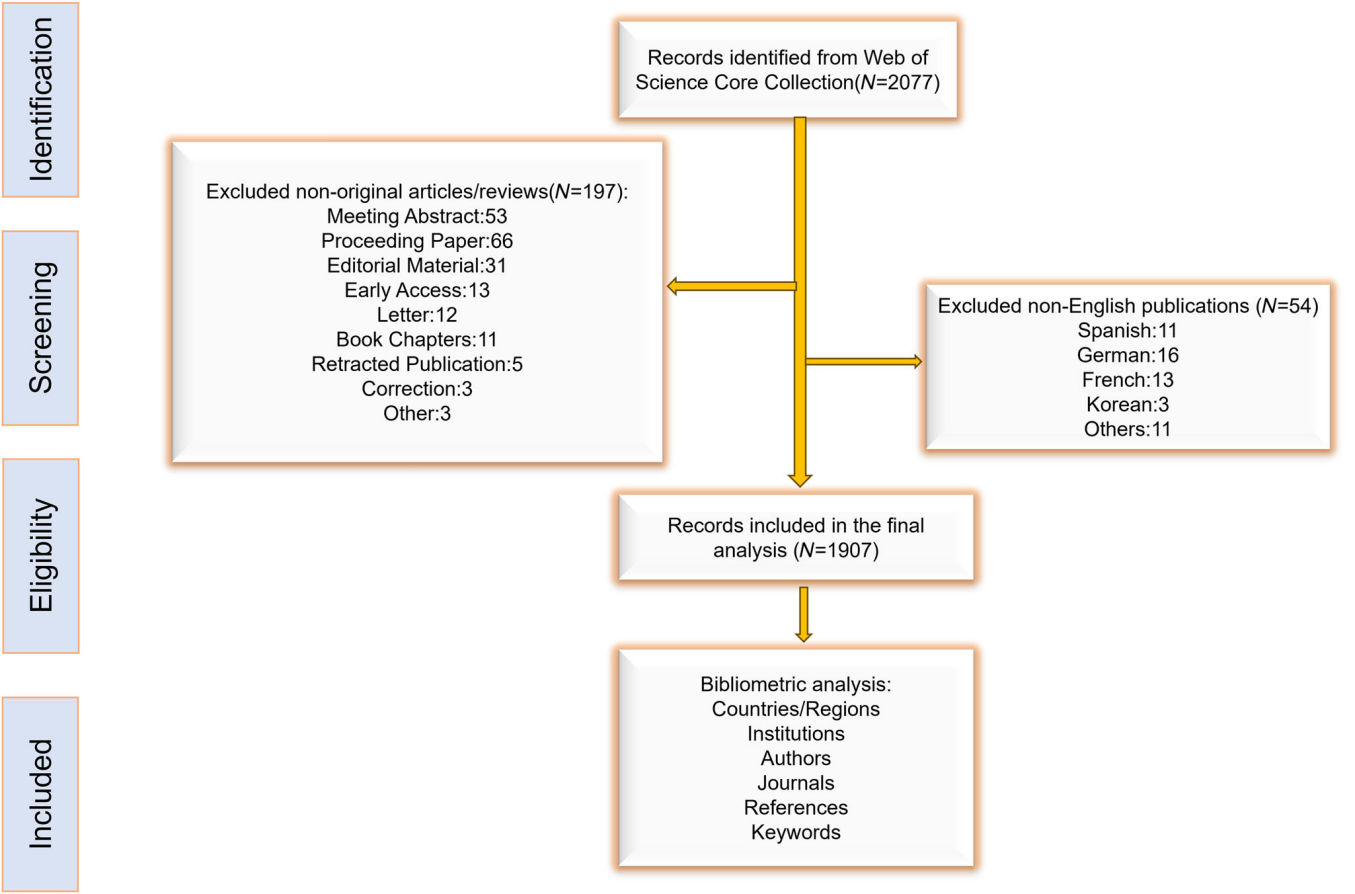
The flowchart of the retrieval strategy and analysis.

The Web of Science Core Collection (WoSCC) (Yi et al. 2025), which indexes more than 18,000 journals across 256 disciplines, is recognized as one of the most authoritative citation databases and is widely applied in bibliometric studies. To reduce potential bias arising from database updates, the literature search was conducted on September 6, 2025. The search strategy was as follows: [TS = (“Cognitive Dysfunctions” OR “Cognitive Dysfunction” OR “Dysfunctions, Cognitive” OR “Cognitive Disorder” OR “Dysfunction, Cognitive” OR “Disorder, Cognitive” OR “Disorders, Cognitive” OR “Cognitive Disorders” OR “Cognitive Impairment” OR “Impairment, Cognitive” OR “Cognitive Impairments” OR “Mild Cognitive Impairment” OR “Impairments, Cognitive” OR “Impairment, Mild Cognitive” OR “Cognitive Impairment, Mild” OR “Mild Cognitive Impairments” OR “Impairments, Mild Cognitive” OR “Cognitive Declines” OR “Cognitive Decline” OR “Declines, Cognitive” OR “Decline, Cognitive” OR “Deterioration, Mental” OR “Deteriorations, Mental” OR “Mental Deteriorations” OR “Mental Deterioration”) AND TS = (“Donepezil” OR “Donepezil Hydrochloride” OR “1‐Benzyl‐4‐((5,6‐dimethoxy‐1‐indanon)‐2‐yl)methylpiperidine hydrochloride” OR “Aricept” OR “E 2020” OR “E‐2020” OR “E2020” OR “Eranz” OR “Donepezilium Oxalate Trihydrate”)]. This search strategy utilizes the PubMed Medical Subject Headings (MeSH) to guarantee thorough and standardized coverage of terminology. The search was restricted to publications in English, and only original articles and review papers were included. A comprehensive search was performed to retrieve all currently available studies in this field. A total of 1907 publications were ultimately included, comprising 1557 original research articles and 350 review papers. The inclusion criteria were as follows: (1) studies focusing on donepezil in the treatment of CI; (2) original research articles or review papers; and (3) publications in English. The exclusion criteria were: (1) studies not primarily addressing donepezil for the treatment of CI; (2) non‐English publications; and (3) papers other than original articles or reviews. The detailed workflow is presented in Figure 1.

### 2.2 Data Analysis and Visualization

CiteSpace focuses on the temporal sequence and evolution of network structures, making it ideal for uncovering key trends and historical developments within a discipline (Yang et al. 2026). In contrast, VOSviewer specializes in group clustering and visualization analysis, clearly illustrating academic groups and collaboration networks within a research field (Tang et al. 2023). Combining these tools offers more in‐depth and comprehensive analytical results, enhancing our understanding of research dynamics and academic trends in the field. Both VOSviewer and CiteSpace were utilized for a thorough analysis and visualization of carefully selected literature. The key analytical elements included country/region, institution, author, journal, funding agency, citation count, and keywords. Publication trends were analyzed using Microsoft Word 2021. After standardizing by country, Scimago Graphica was employed to create a geographical distribution map of publication volume. Finally, the h‐index of authors in the field was calculated using the Bibliometrix software package.

## 3 Results

### 3.1 Global Trends in Annual and Cumulative Publications

**FIGURE 2:**
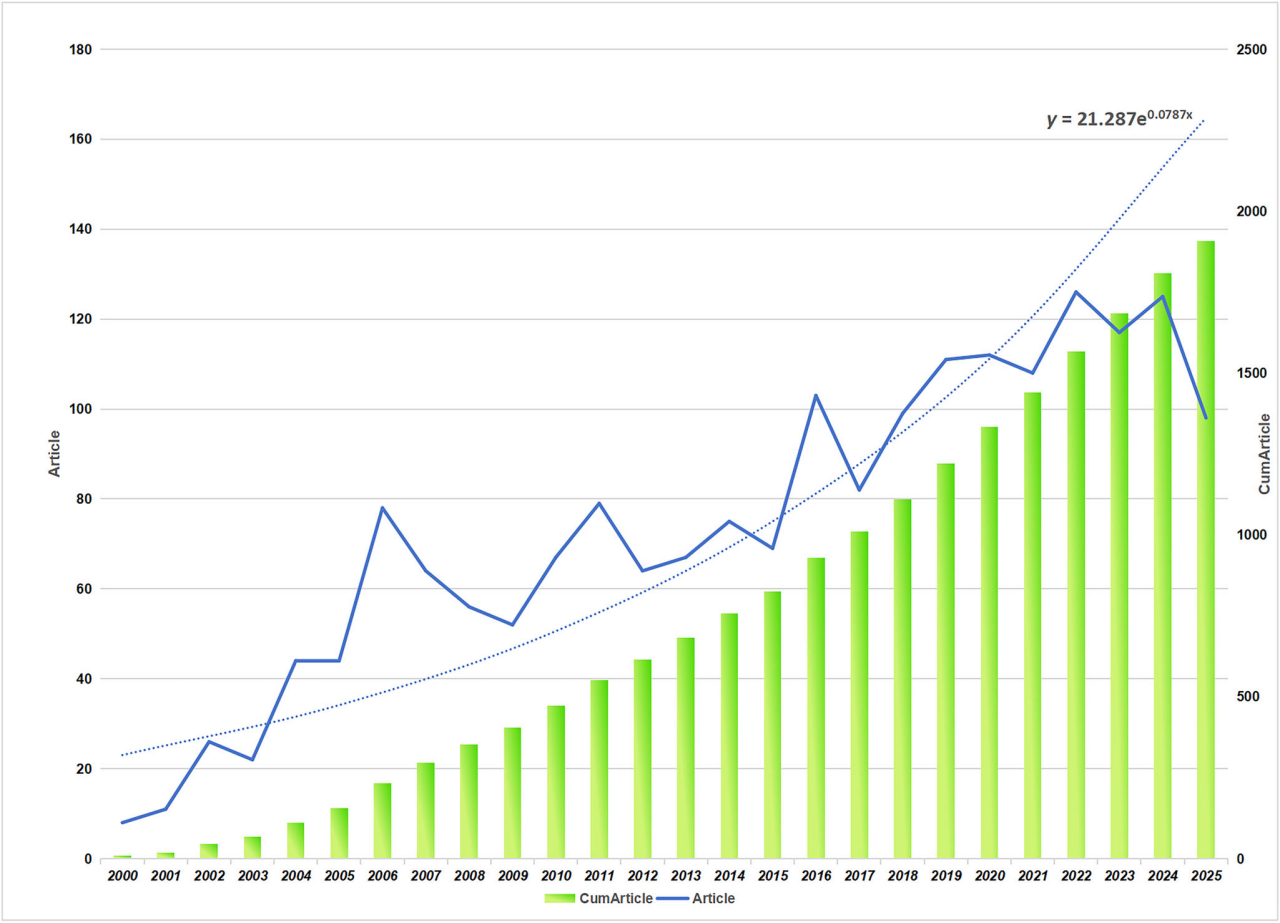
The annual number and the cumulative number of publications.

As shown in Figure 2, a total of 1907 publications were included in the analysis from 2000 to 2025. Over the past 25 years, both annual and cumulative publication counts in this field have demonstrated a sustained upward trajectory, peaking at 126 publications in 2022. Notably, a significant increase was observed between 2005–2006 and 2015–2016, suggesting the emergence of new research topics such as “oxidative stress” and “neostigmine mechanisms” during these periods. Overall, this trend reflects the sustained and rapidly growing scholarly interest in donepezil for the treatment of CI.

### 3.2 Global Distribution and Contributions of Countries/Regions and Institutions

**FIGURE 3:**
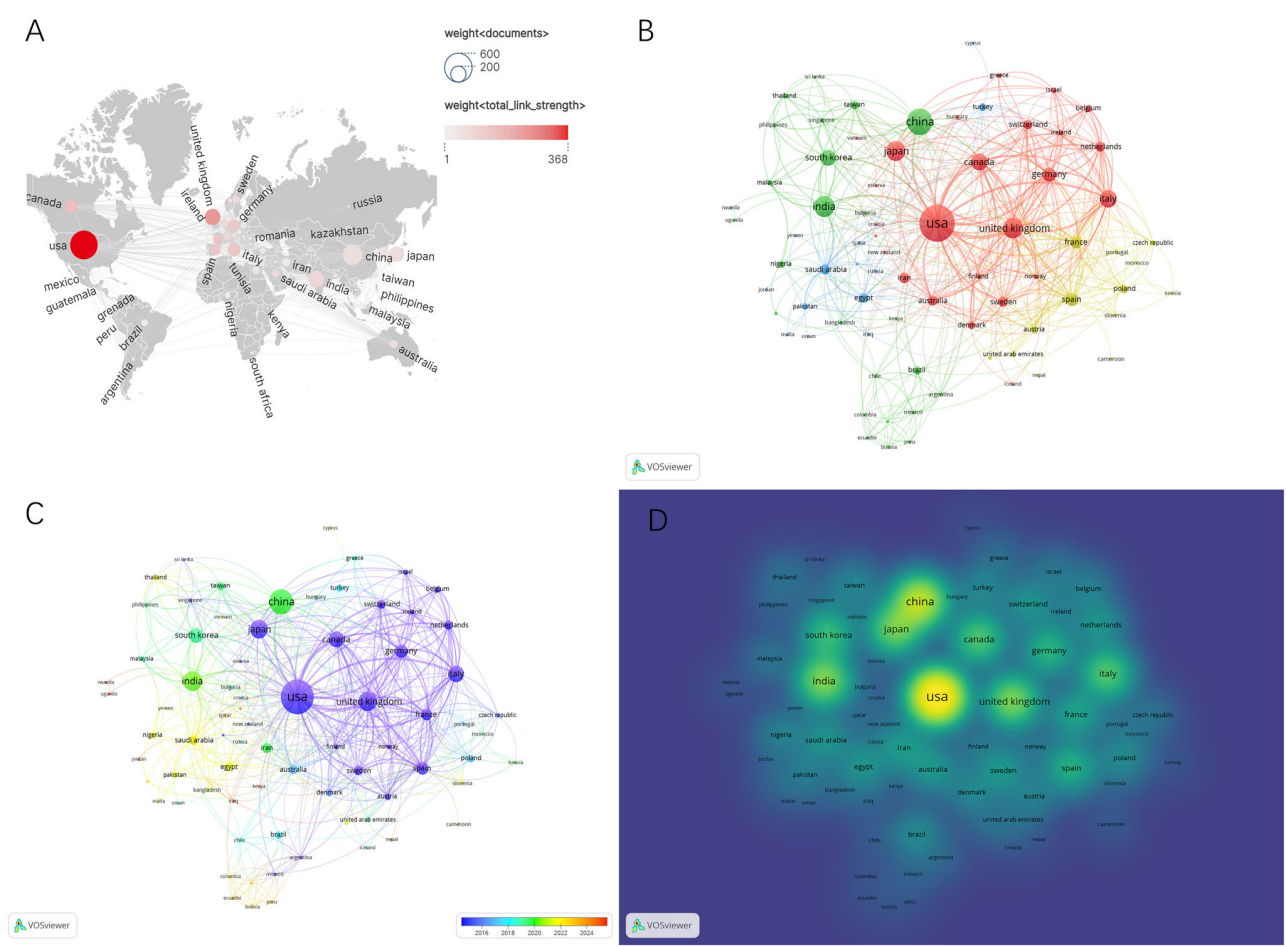
(A) Countries/regions collaboration map. (B) The clustering analysis of the countries/regions. (C) The time‐overlapping visualization of countries/regions collaboration. (D) The heatmap of countries/regions collaboration.

A total of 81 countries/regions have contributed to research on donepezil in the treatment of CI. As demonstrated in Table 1, the United States ranks first with 539 publications (28.3%) and 32,659 citations, followed by China (273 publications, 14.3%; 5861 citations), India (181 publications, 9.5%; 3982 citations), the United Kingdom (170 publications, 8.9%; 10,980 citations), and Japan (157 publications, 8.2%; 3883 citations). Each of the remaining countries contributed fewer than 150 publications. Visualization of the collaboration network using CiteSpace and VOSviewer highlights the dominant positions of the United States and China. Figure 3A illustrates the global collaboration network on a world map, emphasizing the close international partnerships led by China and the United States. Figure 3B depicts a cluster analysis, dividing countries into four major clusters. The largest cluster (red) includes 25 countries, with Canada, the United Kingdom, Japan, Germany, and the United States as central members. Figure 3C presents a timeline of country‐level research activity, showing that contributions have gradually expanded from developed nations such as the United Kingdom and the United States to emerging countries including Rwanda, Uganda, and Jordan. Figure 3D displays a country‐level heatmap. Collectively, these results demonstrate that China and the United States play leading roles in scientific output and international collaboration in this field, driving progress in research on donepezil for the treatment of CI.

**TABLE 1:** Top 10 most publication countries/regions related to cognitive dysfunction and donepezil.

Rank	Country	Articles	Freq. = Articles/total articles	Citations	Average article citations
1	USA	539	28.3%	32,659	60.59
2	China	273	14.3%	5861	21.47
3	India	181	9.5%	3982	22
4	UK	170	8.9%	10,980	64.59
5	Japan	157	8.2%	3883	24.73
6	Italy	125	6.6%	6897	55.18
7	Canada	120	6.3%	5959	49.66
8	South Korea	99	5.2%	2065	20.86
9	Germany	82	4.3%	3853	46.99
10	Spain	76	4.0%	3596	47.32

**FIGURE 4:**
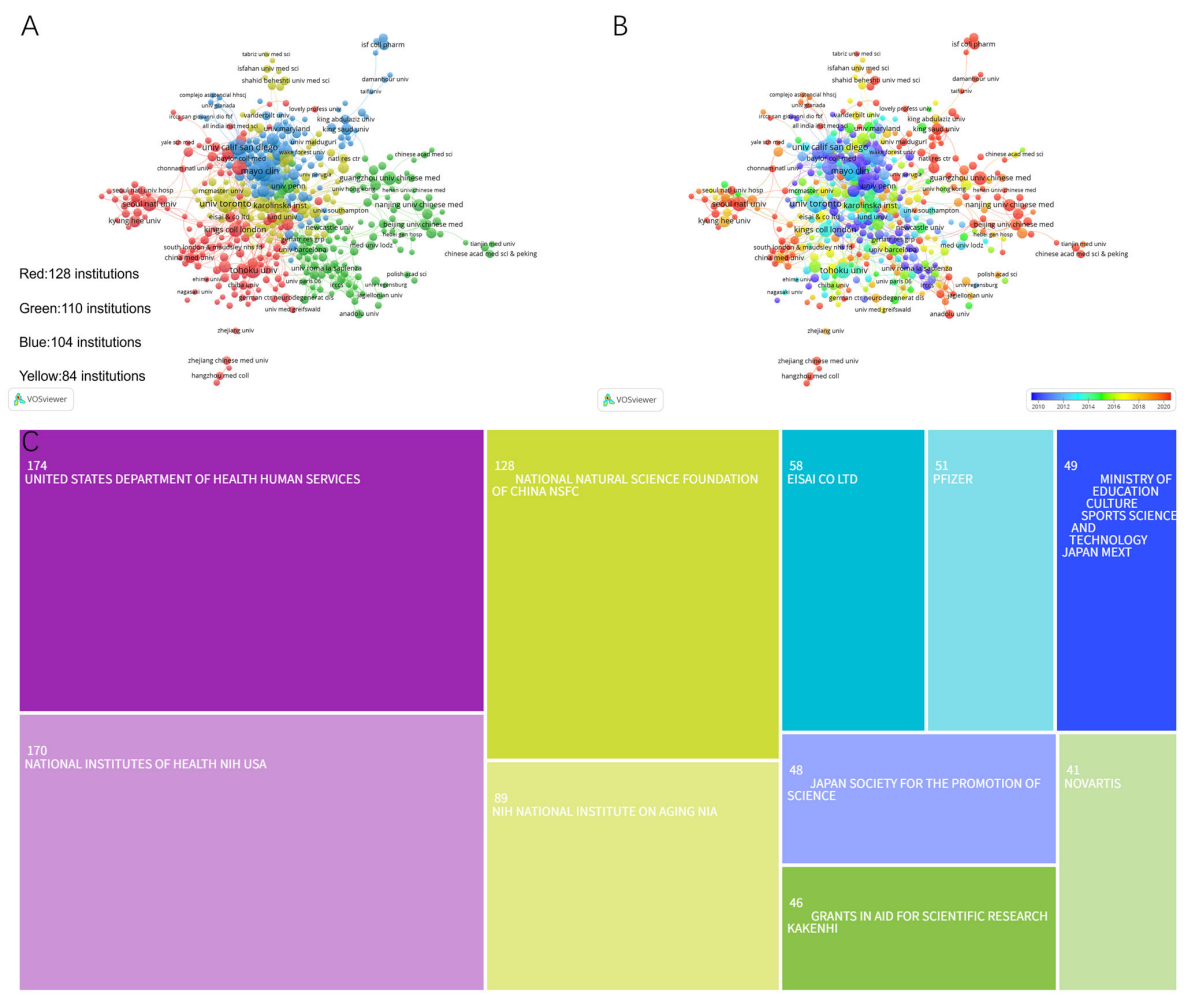
(A) Clustering network and (B) time‐overlapping visualization for institutions. (C) Top 10 funding agencies.

Over the past 25 years, a total of 2909 institutions worldwide have contributed to research on donepezil in the treatment of CI. As shown in Table 2, the University of Toronto (Canada) ranks first in publication volume with 31 papers and 1254 citations. The University of California, Los Angeles (the United States) ranks fourth with 26 publications, yet leads in citations (4300) and average citations per article (165.38). Pfizer Inc. (the United States) ranks first in total link strength (92). Among the top ten institutions, five are from the United States, two from Canada, two from Japan, and one from the United Kingdom, underscoring their pivotal roles in this field. Figure 4A illustrates the institutional collaboration network generated by VOSviewer, which is divided into four major clusters. The largest cluster (red), centered on King's College London, encompasses 128 institutions, highlighting their central influence in this research domain. Figure 4B presents the institutional timeline, showing that research activity has gradually shifted from institutions such as the University of California, Los Angeles, and Brown University to those including Seoul National University and Cairo University.

**TABLE 2:** Top 10 most publication institutions related to cognitive dysfunction and donepezil.

Rank	Institutions	Country/region	Documents	Citations	Average article citations	Total link strength
1	University of Toronto	Canada	31	1254	40.45	79
2	Mayo Clinic	USA	27	3192	118.22	73
3	University of California, San Diego	USA	26	3680	141.54	75
4	University of California, Los Angeles	USA	26	4300	165.38	74
5	Pfizer Inc.	USA	24	1628	67.83	92
6	Tohoku University	Japan	24	527	21.96	37
7	Eisai Inc.	Japan	22	1138	51.73	77
8	McGill University	Canada	22	899	40.86	43
9	Brown University	USA	21	1537	73.19	51
10	King's College London	UK	20	1477	73.85	53

### 3.3 Financial Support in This Field

Figure 4C presents the funding distribution of the included studies. The United Nations Department of Health and Human Services was the largest funding body, supporting 174 projects. The U.S. National Institutes of Health ranked second with 170 funded projects, followed by the National Natural Science Foundation of China with 128 projects. Collectively, these three organizations provided support for more than 50% of the total funded studies, underscoring their central role in advancing research on donepezil for the treatment of CI. Their substantial contributions highlight strong institutional recognition of the future potential of this research field.

### 3.4 Analysis of Journals

At present, a total of 647 journals have published studies on donepezil for the treatment of CI. As shown in Table 3, the *Journal of Alzheimer's Disease* ranks first with 62 publications, followed by *Frontiers in Pharmacology* (31 publications) and *Current Alzheimer Research* (28 publications). Among the top ten journals by publication volume, six belong to the Q1 category and two to the Q2 category. In terms of academic impact, *Neurology* leads with 2601 citations and also ranks first in co‐citations, underscoring its pivotal role in shaping research hotspots and directions in this field.

**TABLE 3:** Top 10 journals and co‐cited journals related to cognitive dysfunction and donepezil.

Rank	Journal	IF (2024)	JCR quantile	Documents	Citations	Co‐cited journal	IF (2024)	JCR quantile
1	*Journal of Alzheimer's Disease*	3.1	Q2	62	1666	*Neurology*	8.5	Q1
2	*Frontiers in Pharmacology*	4.8	Q1	31	562	*Journal of Alzheimer's Disease*	3.1	Q2
3	*Current Alzheimer Research*	1.9	Q3	28	842	*Archives of Neurology (Chicago)*	0	—
4	*International Journal of Geriatric Psychiatry*	2.8	Q1	26	1297	*International Journal of Geriatric Psychiatry*	2.8	Q1
5	*Alzheimer's & Dementia*	11.1	Q1	24	1139	*Alzheimer's & Dementia*	11.1	Q1
6	*Dementia and Geriatric Cognitive Disorders*	1.6	Q3	24	1124	*Dementia and Geriatric Cognitive Disorders*	1.6	Q3
7	*Neurology*	8.5	Q1	21	2601	*Lancet*	88.5	Q1
8	*Alzheimer's Research & Therapy*	7.6	Q1	19	763	*Journal of Neuroscience*	4	Q1
9	*Psychopharmacology*	3.3	Q2	18	991	*American Journal of Psychiatry*	14.7	Q1
10	*Neuropharmacology*	4.6	Q1	18	916	*Journal of Neurology, Neurosurgery and Psychiatry*	7.5	Q1

**FIGURE 5:**
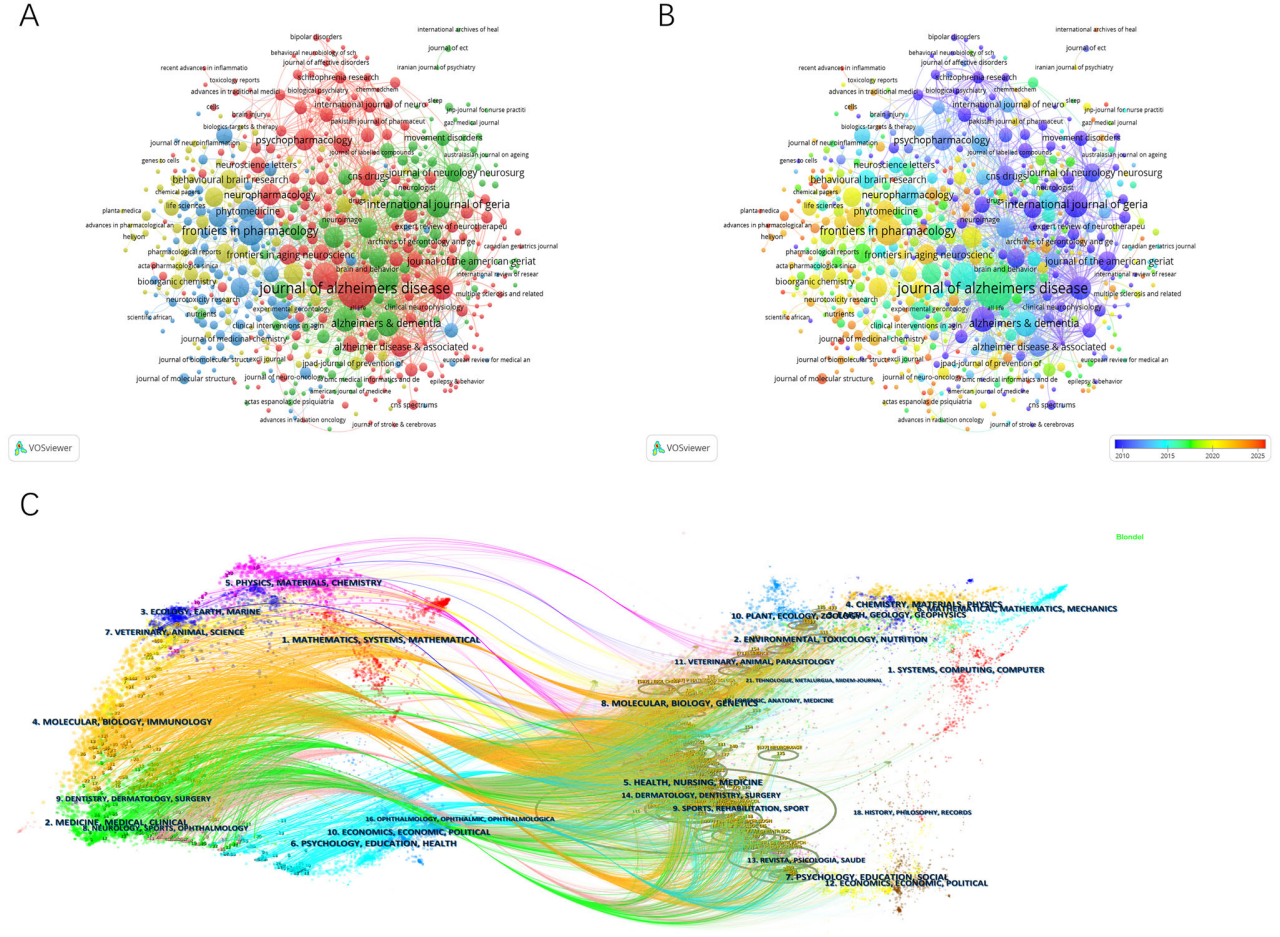
(A) Clustering network and (B) time‐overlapping visualization for the co‐cited journals. (C) Related fields of cognitive dysfunction and donepezil, the left is the literature included in this study, and the right is the reference of this literature.

Using VOSviewer, we further visualized the collaborative journal network in this field. As illustrated in Figure 5A, the journals were grouped into four major clusters: the red cluster, centered on the *Journal of Alzheimer's Disease* and *Psychopharmacology*; the yellow cluster, centered on *Behavioral Brain Research* and *Bioorganic Chemistry*; the green cluster, centered on *Alzheimer's & Dementia* and the *International Journal of Geriatric Psychiatry*; and the blue cluster, centered on *Frontiers in Pharmacology* and *Phytomedicine*. Figure 5B presents a timeline of journals over the past 25 years, showing a gradual shift from outlets such as *Alzheimer's & Dementia* and the *International Journal of Geriatric Psychiatry* to journals including *ChemistrySelect* and the *Journal of Molecular Structure*.

As illustrated in Figure 5C, the relevant literature spans multiple disciplines, including medicine, biology, molecular science, and pharmacology. Specifically, neurology emphasizes the diagnosis and treatment of CI; molecular biology and immunology address the mechanisms of donepezil on the neurotransmitter system, involving molecular‐level regulation and immune‐related pathways; psychology, education, and health sciences contribute to the assessment of psychological status, cognitive function changes, and rehabilitation training in patients with CI; while pharmacology and medicinal chemistry focus on drug structure, pharmacological activity, and pharmacokinetics of donepezil. Such interdisciplinary collaborations represent a major driving force in advancing the effective application of donepezil in the treatment of CI and fostering innovation in both diagnosis and therapy.

### 3.5 Analysis of Author Publication Volume and Collaboration

Over the past 25 years, a total of 9858 authors have contributed publications on donepezil for the treatment of CI. As shown in Table 4, among the most prolific authors, Etsuro Mori ranks first with 12 publications (0.63% of the total) and an h‐index of 10. Ronald C. Petersen and Serge Gauthier both rank second with 11 publications each (0.58%), with h‐indices of 16 and 18, respectively. Manabu Ikeda ranks third with 10 publications (0.52%) and an h‐index of 7. In terms of academic impact, Ronald C. Petersen ranks first with 887 citations, underscoring his significant contributions to the advancement of donepezil research in CI.

**TABLE 4:** Top 10 authors and citation authors related to cognitive dysfunction and donepezil.

Rank	Author	Country	Count	h‐index	Citations	Citation author	Citations
1	Mori, Etsuro	Japan	12 (0.63%)	10	234	Petersen, Ronald C.	593
2	Petersen, Ronald C.	USA	11 (0.58%)	16	887	Winblad, B.	464
3	Gauthier, Serge	Canada	11 (0.58%)	18	377	Cummings, Jeffrey L.	434
4	Ikeda, Manabu	Japan	10 (0.52%)	7	135	Rogers, S. L.	415
5	Pelton, Gregory H.	USA	9 (0.47%)	8	167	Folstein, M. F.	334
6	Singh, Nirmal	India	9 (0.47%)	7	178	Doody, R. S.	294
7	Wang, Qi	China	9 (0.47%)	11	219	Birks, J.	285
8	Babiloni, Claudio	Italy	9 (0.47%)	11	481	Tariot, P. N.	276
9	Cummings, Jeffrey L.	USA	9 (0.47%)	14	490	Aarsland, D.	268
10	Aisen, Paul S.	USA	8 (0.42%)	9	277	Schneider, L. S.	215

**FIGURE 6:**
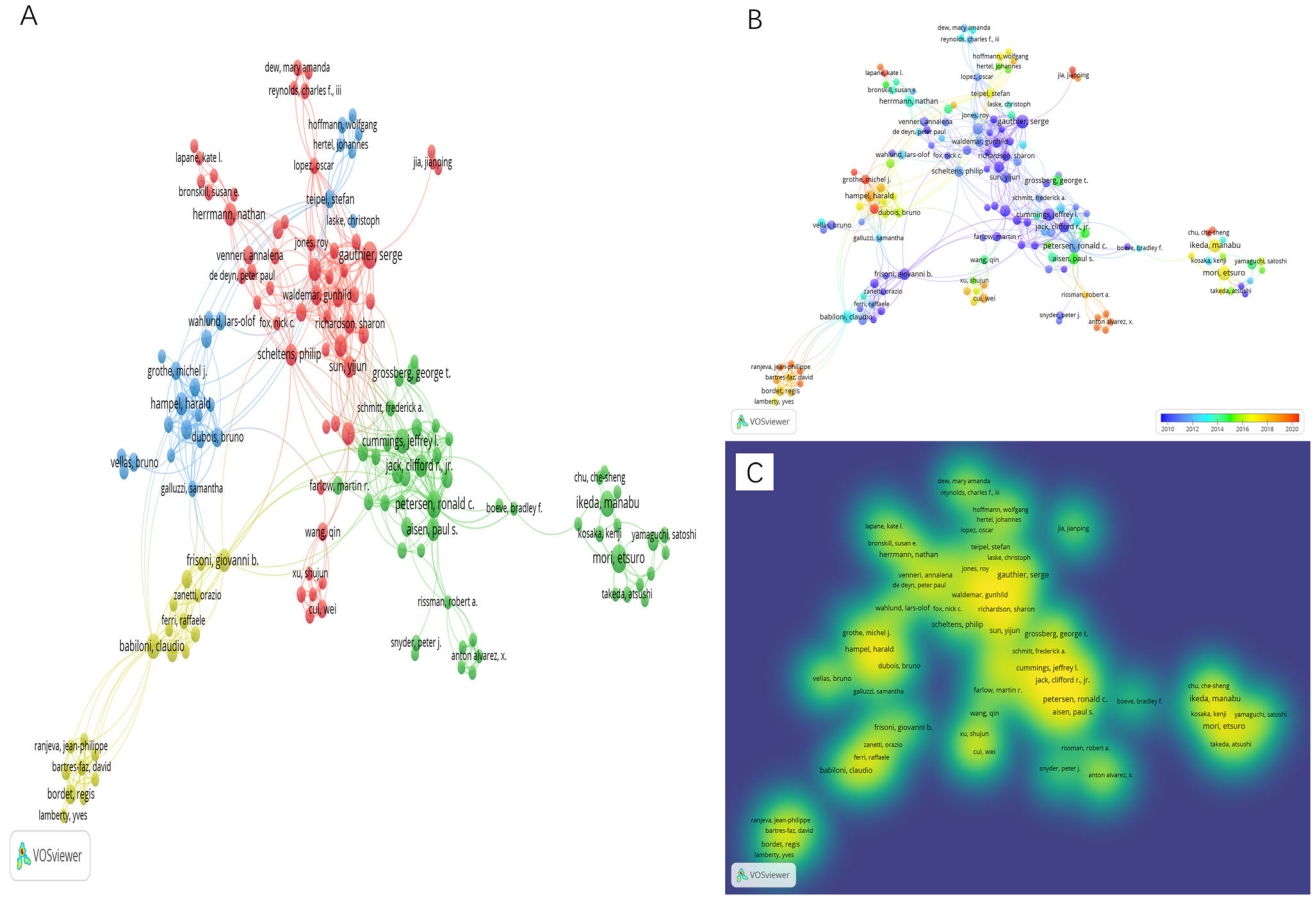
(A) Clustering network, (B) time‐overlapping, and (C) heatmap visualization for the co‐authors.

Figure 6 presents the author collaboration network over the past 25 years. As shown in Figure 6A, authors are divided into four major clusters: the red cluster, centered on Serge Gauthier; the green cluster, centered on Ronald C. Petersen; the blue cluster, centered on Harald Hampel; and the yellow cluster, centered on Claudio Babiloni, reflecting extensive cross‐team collaborations in the field. Figure 6B depicts the evolution of research contributions, showing a transition from early leaders such as Serge Gauthier to more recent emerging contributors, including Michel J. Grothe. Figure 6C displays an author density heatmap, visually demonstrating varying levels of research activity across different contributors.

### 3.6 Co‐Citation and Citation Burst Analysis

File S1 lists the top ten most cited publications among the 1907 articles included in this analysis. The most cited paper is “The clinical use of structural MRI in Alzheimer disease” by Giovanni B. Frisoni et al., published in *Nature Reviews Neurology* in 2010 (Frisoni et al. 2010), with 1476 citations. This is followed by “Vitamin E and donepezil for the treatment of mild cognitive impairment” by Ronald C. Petersen, released in *The New England Journal of Medicine* in 2005, with 1382 citations (Petersen et al. 2005), and “Diagnosis and management of dementia: review” by Zoe Arvanitakis, published in *JAMA* in 2019 (Arvanitakis et al. 2019), with 855 citations.

**FIGURE 7:**
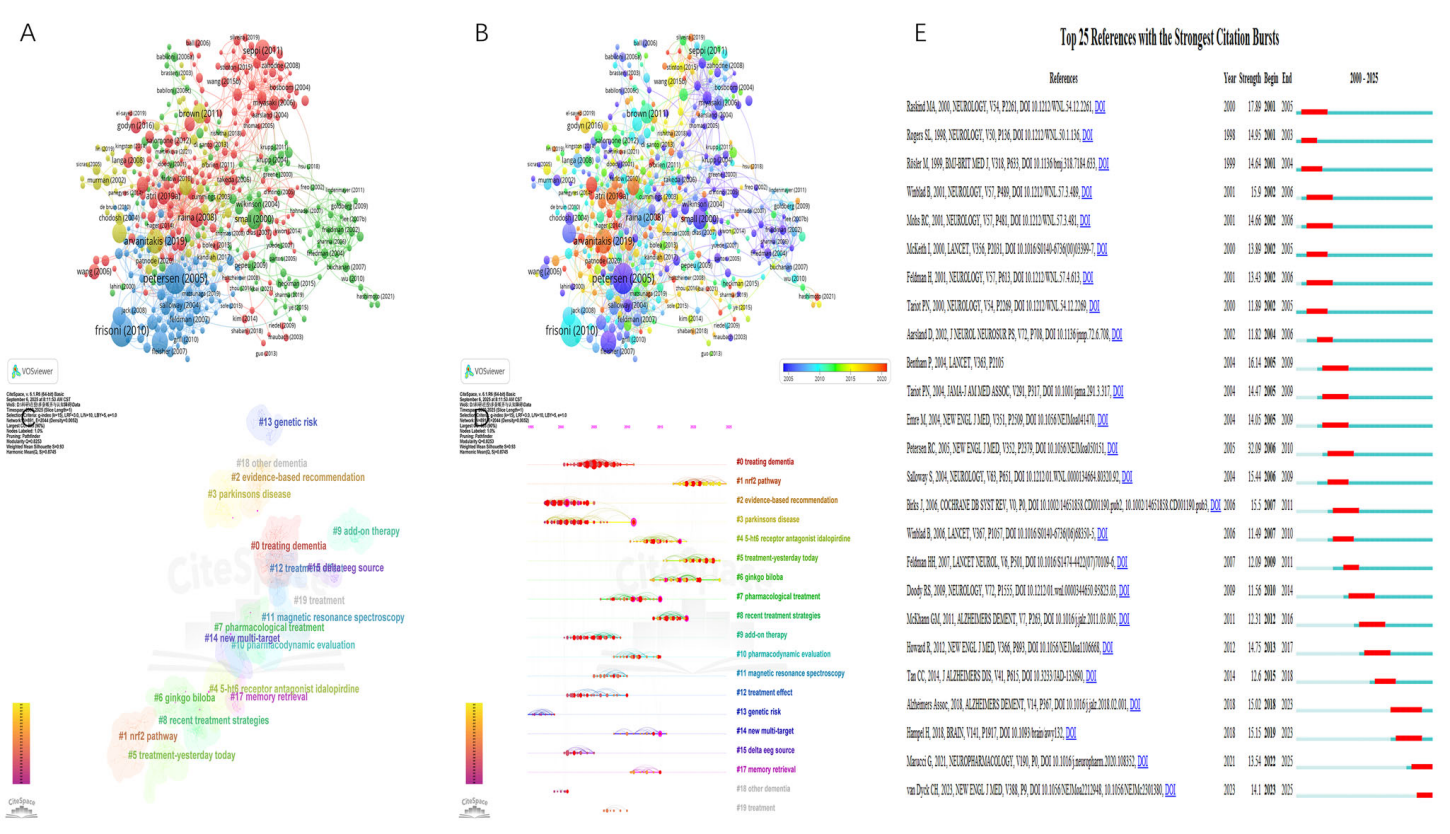
(A) The clustering of references by VOSviewer. (B) The time‐overlapping visualization of references by VOSviewer. (C) The clustering of references by CiteSpace. (D) Timeline of references by CiteSpace. (E) The top 25 references with the highest outbreak intensity.

Figure 7A presents a VOSviewer‐based citation cluster analysis of research on donepezil for the treatment of CI, dividing all citations into four major clusters. Figure 7B illustrates a timeline visualization of these citations. Similarly, Figure 7C,D displays the citation clusters and timeline visualizations generated by CiteSpace. Overall, the citations can be grouped into three broad thematic areas. The first theme relates to disease treatment, including clusters such as “#0 treating dementia,” “#3 Parkinson's disease,” “#7 pharmacological treatment,” “#8 recent treatment strategies,” “#9 add‐on therapy,” “#12 treatment effect,” and “#19 treatment.” The second theme focuses on molecular mechanisms and drug efficacy evaluation, encompassing “#1 Nrf2 pathway,” “#10 pharmacodynamic evaluation,” “#11 magnetic resonance spectroscopy,” and “#13 genetic risk.” The third theme pertains to drug discovery and multi‐target exploration, including “#4 5‐HT6 receptor antagonist idalopirdine,” “#6 Ginkgo biloba,” and “#14 new multi‐target.” Figure 7E highlights the top 25 references with the strongest citation bursts. The article “Vitamin E and donepezil for the treatment of mild cognitive impairment” by Petersen et al. (2005), published in 2005, ranks first with a burst intensity of 32.09. Notably, two studies—“Efficacy of acetylcholinesterase inhibitors in Alzheimer's disease” by Marucci et al. (2021) and “Lecanemab in early Alzheimer's disease” by van Dyck et al. (2023)—continued to show strong citation bursts in 2025, underscoring their sustained influence on donepezil research in CI treatment.

### 3.7 Analysis of Keyword Co‐Occurrence

Using VOSviewer, we analyzed 6483 keywords extracted from 2109 articles. As shown in Table 5, “Alzheimer's disease” ranked first with 1233 occurrences, followed by “Donepezil” (1010) and “Dementia” (595). Other frequently occurring keywords (≥ 200 occurrences) included “Cognitive impairment,” “Double‐blind,” “Mild cognitive impairment,” “Cholinesterase‐inhibitors,” “Memory,” “Efficacy,” and “Rivastigmine.”

**TABLE 5:** Top 20 keywords related to cognitive dysfunction and donepezil.

Rank	Keyword	Occurrences	Total link strength	Rank	Keyword	Occurrences	Total link strength
1	Alzheimer's disease	1233	9571	11	Oxidative stress	199	1563
2	Donepezil	1010	7944	12	Placebo‐controlled trial	169	1580
3	Dementia	595	5046	13	Memantine	167	1559
4	Cognitive impairment	393	3131	14	Galantamine	154	1466
5	Double‐blind	376	3426	15	Cognition	144	1276
6	Mild cognitive impairment	364	3088	16	Acetylcholinesterase	143	1160
7	Cholinesterase‐inhibitors	268	2513	17	Diagnosis	139	1156
8	Memory	246	2001	18	Brain	137	1091
9	Efficacy	235	2179	19	Vascular dementia	115	1028
10	Rivastigmine	200	1921	20	Impairment	114	983

**FIGURE 8:**
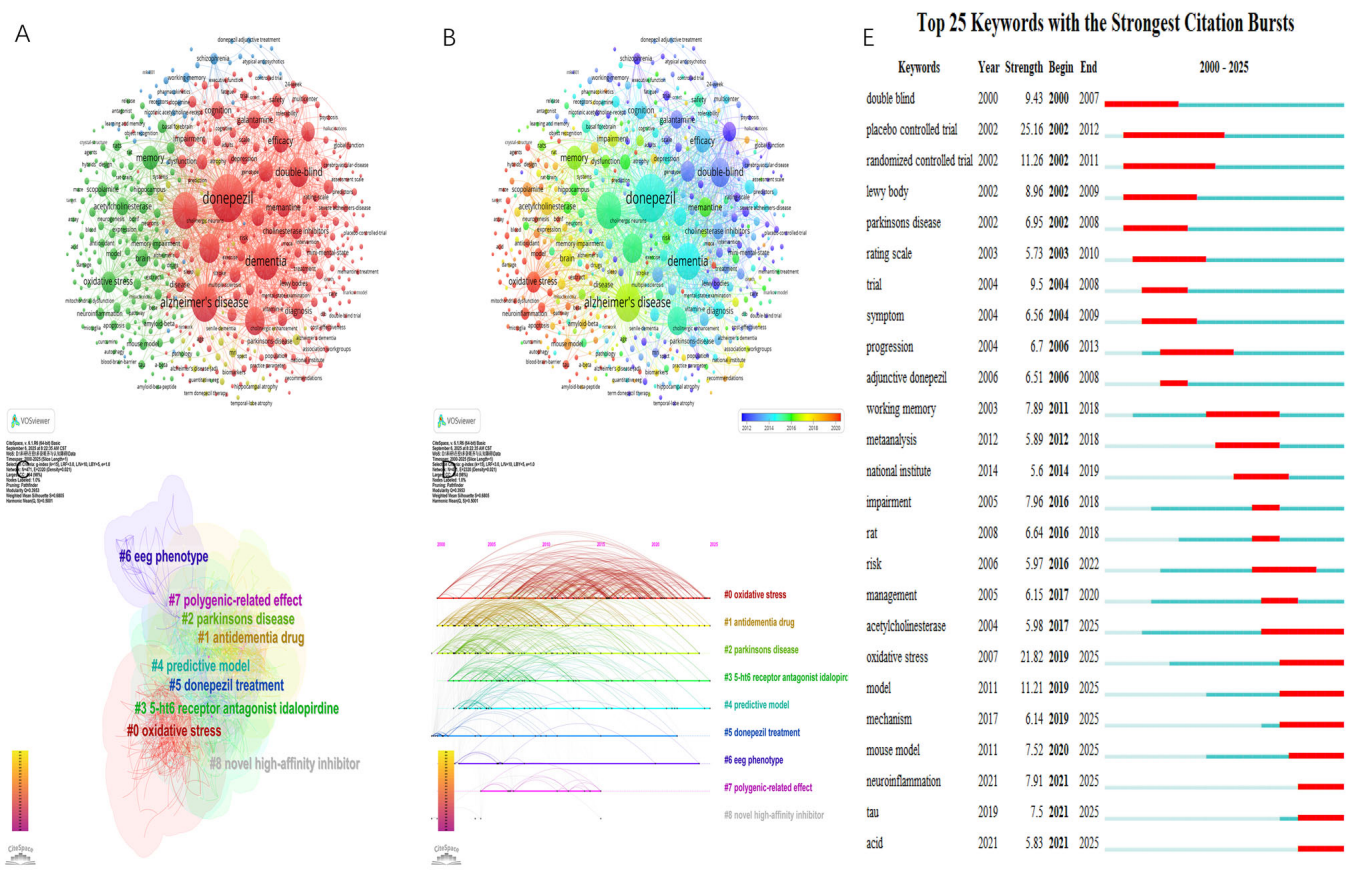
(A) The clustering of keywords by VOSviewer. (B) The time‐overlapping visualization of keywords by VOSviewer. (C) The clustering of keywords by CiteSpace. (D) Timeline of keywords by CiteSpace. (E) The top 25 keywords with the highest outbreak intensity.

Figure 8A presents the keyword cluster analysis generated by VOSviewer, which categorizes all keywords into four major clusters. Figure 8B illustrates the VOSviewer keyword timeline, clearly demonstrating the evolution of research terminology—from early terms such as “double‐blind” and “galantamine” to more recent terms including “oxidative stress” and “neuroinflammation,” which directly reflect current research frontiers in this field. Figure 8C displays the keyword clusters generated by CiteSpace, identifying nine distinct groups. As shown in Figure 8D, the CiteSpace keyword timeline highlights a gradual shift in research focus: earlier studies emphasized disease and drug foundations (e.g., #1 anti‐dementia drugs, #2 Parkinson's disease) and pathological mechanisms (e.g., #0 oxidative stress), while more recent studies have focused on precision medicine and predictive modeling (e.g., #4 predictive models), in‐depth evaluation of donepezil therapy (e.g., #5 donepezil therapy), and emerging directions such as #6 EEG phenotypes, #7 polygenic effects, and #8 novel high‐affinity inhibitors. Figure 8E lists the top 25 keywords with the strongest citation bursts. “Placebo‐controlled trial” showed the highest burst intensity (25.16). Notably, eight keywords—“acetylcholinesterase,” “oxidative stress,” “model,” “mechanism,” “mouse model,” “neuroinflammation,” “tau,” and “acid”—have continued to display strong citation bursts up to the present, underscoring their importance as emerging research hotspots.

## 4 Discussion

### 4.1 Global Knowledge Structure

In the field of donepezil research for the treatment of CI, the number of publications has shown steady fluctuations over the past 25 years. The United States has produced nearly twice as many publications as China, and together the two countries account for approximately 43% of the total output. With respect to institutional contributions, most leading institutions are based in the United States, while none of the top ten publishing institutions are from China. Similarly, the majority of high‐contributing authors are affiliated with U.S. institutions. Substantial research funding from both the United States and China has further fueled progress in this field. Moreover, the journals in which these studies are published and cited are of high quality, with most belonging to the JCR Q1 category. This study highlights that developed countries like the United States and Canada lead in both research output and impact, likely due to their focus on major health issues such as CI. Their success is driven by national research strategies, stable funding, strong infrastructure, and a culture that promotes innovation and collaboration. In addition, their healthcare systems benefit from talent, technology, and drug accessibility, supporting high‐quality research. Future research could explore how transnational collaboration and resource allocation can optimize global research output and impact.

### 4.2 Global Research Priorities and Trends

Further analysis of the most highly cited papers provides valuable insights into hot topics and emerging trends in research on donepezil for the treatment of CI. Overall, the top ten most cited studies can be grouped into four main categories. The first category is AD‐related research, including studies such as “The clinical use of structural MRI in Alzheimer disease” by Frisoni et al. (2010), “Cerebral metabolic and cognitive decline in persons at genetic risk for Alzheimer's disease” by Small et al. (2000), and “MRI of hippocampal volume loss in early Alzheimer's disease in relation to ApoE genotype and biomarkers” by Schuff et al. (2009). The second category is diagnosis, treatment, and management of dementia, represented by studies such as “Diagnosis and management of dementia: review” by Arvanitakis et al. (2019), “Effectiveness of cholinesterase inhibitors and memantine for treating dementia: evidence review for a clinical practice guideline” by Raina et al. (2008), and “Effectiveness of collaborative care for older adults with Alzheimer disease in primary care: a randomized controlled trial” by Callahan et al. (2006). The third category concerns MCI, including “Vitamin E and donepezil for the treatment of mild cognitive impairment” by Petersen et al. (2005) and “Mild cognitive impairment can be distinguished from Alzheimer disease and normal aging for clinical trials” by Grundman et al. (2004). The fourth category focuses on non‐motor symptoms of PD, represented by W. R. Brown's review “Review: cerebral microvascular pathology in ageing and neurodegeneration” (Brown and Thore 2011). Collectively, these studies encompass the diagnosis, treatment, and pathological mechanisms of neurodegenerative disorders such as AD, dementia, MCI, and PD. They provide an essential foundation for ongoing research and have revealed critical insights into the role of donepezil in the treatment of CI. The top ten most‐cited papers in this field highlight a core research trajectory driven by clinical needs, focusing on AD, MCI, and non‐motor symptoms of PD, with emphasis on diagnosis, treatment, and mechanisms. These papers establish a dual‐driven model of basic mechanisms and clinical translation, while identifying gaps such as limited exploration of non‐cholinergic pathways, insufficient research on medications for specific populations, and a lack of integration of emerging technologies, thus guiding future precision medicine research.

Keyword analysis reveals six core thematic categories in research on donepezil for CI. The first category is the core drug, “Donepezil,” which ranks first with 1010 occurrences and a total link strength of 7944, underscoring its central role in this research field. The second category relates to diseases, including high‐frequency keywords such as “Dementia,” “Alzheimer's disease,” “Mild cognitive impairment,” and “Cognitive impairment.” Their high occurrence and strong link strength reflect the primary research focus on CI‐related disorders. The third category concerns other therapeutic agents and mechanisms, including drugs such as “Memantine,” “Galantamine,” and “Rivastigmine,” as well as mechanistic terms like “Cholinesterase inhibitors,” “Acetylcholinesterase,” and “Cholinesterase‐inhibitors,” indicating an emphasis on related pharmacological agents and mechanisms of action. The fourth category involves study design and evaluation, represented by methodological keywords such as “double‐blind” and “placebo‐controlled trial,” alongside evaluation terms including “Efficacy,” “Cognition,” and “Memory.” These reflect the importance of rigorous experimental design and outcome assessment in the field. The fifth category is pathology‐related, with keywords such as “Oxidative stress,” highlighting the attention given to underlying pathological mechanisms. The sixth category encompasses miscellaneous terms, including “Diagnosis” and “Brain,” reflecting the broader relevance of this research to diagnostic practices and brain‐related aspects of cognitive function. In recent years, emerging concepts such as oxidative stress, neuroinflammation, and polygenic effects have attracted increasing attention in research on donepezil for the treatment of CI. The conventional paradigm, which primarily attributes the therapeutic effects of donepezil to cholinesterase inhibition, is insufficient to explain its limited or variable efficacy in a substantial proportion of patients. In this context, investigations into non‐cholinergic mechanisms—including oxidative stress and neuroinflammatory processes—have provided novel perspectives for addressing this clinical challenge. Concurrently, advances in molecular biology and multi‐omics technologies have enabled the precise profiling of inflammatory mediators and genetic polymorphisms in patients with CI, thereby offering a robust methodological foundation for the exploration of polygenic influences on treatment response. Future studies should therefore emphasize multi‐target synergistic mechanisms, with particular attention to the interplay between oxidative stress pathways and cholinergic signaling. Such efforts may facilitate the development of precision medicine strategies, including the identification of patient subgroups most likely to benefit from donepezil based on inflammatory biomarkers, as well as the design of individualized therapeutic regimens informed by genetic profiling.

### 4.3 Application of Donepezil in the Treatment of CI

Donepezil is a widely used cholinesterase inhibitor for the treatment of various forms of CI. Its primary mechanism of action involves inhibiting acetylcholinesterase in the central nervous system, thereby reducing acetylcholine degradation and increasing acetylcholine levels in the brain. Donepezil is a first‐line therapy for mild to moderate AD and has also been approved for the management of severe AD in certain regions. Experimental evidence further demonstrates that combination therapy with donepezil and nimodipine may ameliorate cognitive deficits in AD, potentially through modulation of the gut microbiome and associated metabolites (J. Liu et al. 2025). Another experimental study demonstrated that febuxostat, either alone or in combination with donepezil, may exert protective effects against scopolamine‐induced AD in rats, potentially through modulation of the TXNIP/NLRP3 inflammasome signaling pathway (Taha et al. 2025). Although not universally approved, several studies have suggested that donepezil may be beneficial for the treatment of MCI. A meta‐analysis reported that a higher dose of donepezil (10 mg) remarkably attenuated hippocampal atrophy in patients with MCI, indicating a potential neuroprotective effect (Ismail et al. 2025). In addition, a meta‐analysis demonstrated that the combined use of oral Chinese medicine and donepezil was more effective than donepezil alone in improving cognitive function in patients with MCI, without increasing the incidence of adverse events (L. Liu et al. 2024). These findings suggest that this combination therapy may represent a preferable option in clinical practice. There is increasing evidence supporting the therapeutic role of donepezil in VaD. A meta‐analysis reported that *Ginkgo* leaf extract combined with donepezil hydrochloride was effective in treating Chinese patients with VaD (Xiao et al. 2024). An experimental study demonstrated that donepezil enhanced brain‐derived neurotrophic factor induction by reducing HDAC6 nuclear translocation, thereby alleviating VaD in rats (Jian et al. 2020). In addition, donepezil is recommended for the management of Parkinson's disease dementia (PDD) and dementia with Lewy bodies (DLB). In patients with DLB, donepezil may alleviate cognitive fluctuations and neuropsychiatric symptoms and appears to be safer than other acetylcholinesterase inhibitors. Furthermore, a meta‐analysis reported that donepezil demonstrated overall efficacy in improving both CI and global clinical status in patients with DLB (Mori, Ikeda, and Ohdake 2024). A randomized controlled trial demonstrated that a 10‐mg dose of donepezil was effective in improving cognitive function in patients with DLB (Mori, Ikeda, Iseki, et al. 2024). Another randomized controlled trial demonstrated that donepezil could help prevent the progression to dementia in patients with PD who exhibited severe olfactory impairment (Baba et al. 2022).

Although donepezil is widely used in the treatment of various types of CI, its therapeutic efficacy has limitations. Not all patients derive benefit, and interindividual differences in response appear to be associated with factors such as disease stage, genetic polymorphisms, and baseline cholinergic function. Current research increasingly emphasizes individualized therapy guided by biomarkers, including cerebrospinal fluid acetylcholine levels and positron emission tomography imaging, to identify potential responders. Combinational therapy has also become a major focus in this field. For example, the combination of donepezil and memantine is increasingly applied in moderate to severe AD, with some clinical trials demonstrating synergistic benefits (Atri et al. 2015). In addition, combination strategies with disease‐modifying drugs, such as the anti‐amyloid antibody lecanemab, are under investigation to simultaneously target both clinical symptoms and underlying pathological mechanisms. Looking ahead, further preclinical and early‐phase clinical studies are warranted to expand the therapeutic potential of donepezil beyond AD, including applications in cognitive decline associated with traumatic brain injury (Youn et al. 2024), Down syndrome‐related dementia (Kishnani et al. 2009), and other forms of CI.

### 4.4 Limitations

Although this study comprehensively reviewed existing research on donepezil for the treatment of CI and summarized emerging research topics and development trends, several limitations should be acknowledged. First, the exclusive reliance on the WoSCC database may have led to the omission of relevant publications, although such omissions are unlikely to substantially affect the overall conclusions. Second, the inclusion criteria, which are restricted to English‐language publications, research papers, and reviews, may overlook valuable studies published in other languages or formats, potentially introducing some bias. Third, the results may have been influenced by the timing and timeframe of the literature search, which could introduce potential bias. Fourth, CiteSpace and VOSviewer are widely used bibliometric tools, but they have limitations, including dependence on data sources, technical deficiencies, inability to reveal biological significance, low sensitivity to emerging trends, and susceptibility to parameter settings and manual preprocessing. Thus, integrating expert knowledge and optimization strategies is essential to improve the rigor of the analysis.

## 5 Conclusion

Over the past 25 years, research on the treatment of CI has developed rapidly, with China and the United States emerging as the leading contributors. The University of Toronto has published the highest number of articles in this field, while the U.S. Department of Health and Human Services has supplied the largest share of funding. The *Journal of Alzheimer's Disease* is the most productive journal, and leading researchers such as Etsuro Mori, Ronald C. Petersen, and Serge Gauthier have played pivotal roles in advancing the field. Future research is expected to emphasize individualized drug administration, combination strategies with disease‐modifying agents, and the extension of therapeutic approaches to underexplored CI subtypes, with the goal of further optimizing the clinical value of donepezil.

## Author Contributions

Research design, data analysis, figure creation, and writing: Wencai Wang. Research design, data analysis, figure creation, writing, review, and editing: Yinuo Chen and Zijie Xiong. Relevant literature collection and writing: Zun Wang and Wei Ye. Research design, review, and funding: Xianfeng Li. All authors have reviewed, revised, read, and approved the final manuscript.

## Funding

This work was supported in part by grants from the Neurosurgery First‐class Discipline Funding Project of the Second Affiliated Hospital of Harbin Medical University.

## Ethics Statement

The authors have nothing to report.

## Consent

The authors have nothing to report.

## Conflicts of Interest

The authors declare no conflicts of interest.

## Supporting information



Supplementary File 1: The top 10 most co‐cited references.

## Data Availability

All the data can be obtained from the open‐source website we provide, and the conclusions can be drawn through analysis with the relevant software.

## References

[brb371251-bib-0001] Arvanitakis, Z. , R. C. Shah , and D. A. Bennett . 2019. “Diagnosis and Management of Dementia: Review.” JAMA 322, no. 16: 1589–1599. 31638686 10.1001/jama.2019.4782 PMC7462122

[brb371251-bib-0002] Atri, A. , S. B. Hendrix , V. Pejović , et al. 2015. “Cumulative, Additive Benefits of Memantine‐Donepezil Combination Over Component Monotherapies in Moderate to Severe Alzheimer's Dementia: A Pooled Area Under the Curve Analysis.” Alzheimer's Research & Therapy 7, no. 1: 28. 10.1186/s13195-015-0109-2 PMC4436119 25991927

[brb371251-bib-0003] Baba, T. , A. Takeda , A. Murakami , T. Koga , T. Isomura , and E. Mori . 2022. “Effect of Donepezil for Dementia Prevention in Parkinson's Disease With Severe Hyposmia (The DASH‐PD Study): A Randomized Long‐Term Placebo‐Controlled Trial.” EClinicalMedicine 51: 101571. 35860451 10.1016/j.eclinm.2022.101571 PMC9289637

[brb371251-bib-0004] Baik, K. , S. M. Kim , J. H. Jung , et al. 2021. “Donepezil for Mild Cognitive Impairment in Parkinson's Disease.” Scientific Reports 11, no. 1: 4734. 33637811 10.1038/s41598-021-84243-4 PMC7910590

[brb371251-bib-0005] Battle, C. E. , A. H. Abdul‐Rahim , S. D. Shenkin , J. Hewitt , and T. J. Quinn . 2021. “Cholinesterase Inhibitors for Vascular Dementia and Other Vascular Cognitive Impairments: A Network Meta‐Analysis.” Cochrane Database of Systematic Reviews 2, no. 2: CD013306. 33704781 10.1002/14651858.CD013306.pub2 PMC8407366

[brb371251-bib-0006] Brown, W. R. , and C. R. Thore . 2011. “Review: Cerebral Microvascular Pathology in Ageing and Neurodegeneration.” Neuropathology and Applied Neurobiology 37, no. 1: 56–74. 20946471 10.1111/j.1365-2990.2010.01139.x PMC3020267

[brb371251-bib-0007] Callahan, C. M. , M. A. Boustani , F. W. Unverzagt , et al. 2006. “Effectiveness of Collaborative Care for Older Adults With Alzheimer Disease in Primary Care: A Randomized Controlled Trial.” JAMA 295, no. 18: 2148–2157. 16684985 10.1001/jama.295.18.2148

[brb371251-bib-0008] Cao, Q. , C. C. Tan , W. Xu , et al. 2020. “The Prevalence of Dementia: A Systematic Review and Meta‐Analysis.” Journal of Alzheimer's Disease 73, no. 3: 1157–1166. 10.3233/JAD-191092 31884487

[brb371251-bib-0009] Diaz‐Galvan, P. , G. Lorenzon , R. Mohanty , et al. 2023. “Differential Response to Donepezil in MRI Subtypes of Mild Cognitive Impairment.” Alzheimer's Research & Therapy 15, no. 1: 117. 10.1186/s13195-023-01253-2 PMC10288762 37353809

[brb371251-bib-0010] Eshkoor, S. A. , T. A. Hamid , C. Y. Mun , and C. K. Ng . 2015. “Mild Cognitive Impairment and Its Management in Older People.” Clinical Interventions in Aging 10: 687–693. 25914527 10.2147/CIA.S73922 PMC4401355

[brb371251-bib-0011] Frisoni, G. B. , N. C. Fox , C. R. Jack Jr. , P. Scheltens , and P. M. Thompson . 2010. “The Clinical Use of Structural MRI in Alzheimer Disease.” Nature Reviews Neurology 6, no. 2: 67–77. 20139996 10.1038/nrneurol.2009.215 PMC2938772

[brb371251-bib-0012] Grundman, M. , R. C. Petersen , S. H. Ferris , et al. 2004. “Mild Cognitive Impairment Can Be Distinguished From Alzheimer Disease and Normal Aging for Clinical Trials.” Archives of Neurology 61, no. 1: 59–66. 14732621 10.1001/archneur.61.1.59

[brb371251-bib-0013] Ismail, Y. A. , Y. Haitham , M. Walid , H. Mohamed , and Y. M. A. El‐Satar . 2025. “Efficacy of Acetylcholinesterase Inhibitors on Reducing Hippocampal Atrophy Rate: A Systematic Review and Meta‐Analysis.” BMC Neurology 25, no. 1: 60. 39939901 10.1186/s12883-024-03933-4 PMC11816531

[brb371251-bib-0014] Jian, W. X. , Z. Zhang , J. H. Zhan , et al. 2020. “Donepezil Attenuates Vascular Dementia in Rats Through Increasing BDNF Induced by Reducing HDAC6 Nuclear Translocation.” Acta Pharmacologica Sinica 41, no. 5: 588–598. 31913348 10.1038/s41401-019-0334-5 PMC7470853

[brb371251-bib-0015] Jongsiriyanyong, S. , and P. Limpawattana . 2018. “Mild Cognitive Impairment in Clinical Practice: A Review Article.” American Journal of Alzheimer's Disease and Other Dementias 33, no. 8: 500–507. 10.1177/1533317518791401 PMC10852498 30068225

[brb371251-bib-0016] Kishnani, P. S. , B. R. Sommer , B. L. Handen , et al. 2009. “The Efficacy, Safety, and Tolerability of Donepezil for the Treatment of Young Adults With Down Syndrome.” American Journal of Medical Genetics Part A 149A, no. 8: 1641–1654. 19606472 10.1002/ajmg.a.32953

[brb371251-bib-0017] Liu, J. , K. Liu , X. Cui , et al. 2025. “From Gut to Brain: Donepezil and Nimodipine Combination Therapy Improves Cognitive Deficits in Alzheimer's Disease via Gut Microbiota and Metabolites.” Biochemical and Biophysical Research Communications 778: 152418. 40737743 10.1016/j.bbrc.2025.152418

[brb371251-bib-0018] Liu, L. , C. S. Zhang , A. L. Zhang , Y. Cai , and C. C. Xue . 2024. “Oral Chinese Herbal Medicine Combined With Donepezil for Mild Cognitive Impairment: A Systematic Review and Meta‐Analysis.” Journal of the American Geriatrics Society 72, no. 12: 3890–3902. 39134455 10.1111/jgs.19125 PMC11637298

[brb371251-bib-0019] Lopez, O. L. , and L. H. Kuller . 2019. “Epidemiology of Aging and Associated Cognitive Disorders: Prevalence and Incidence of Alzheimer's Disease and Other Dementias.” Handbook of Clinical Neurology 167: 139–148. 31753130 10.1016/B978-0-12-804766-8.00009-1

[brb371251-bib-0020] Marucci, G. , M. Buccioni , D. D. Ben , C. Lambertucci , R. Volpini , and F. Amenta . 2021. “Efficacy of Acetylcholinesterase Inhibitors in Alzheimer's Disease.” Neuropharmacology 190: 108352. 33035532 10.1016/j.neuropharm.2020.108352

[brb371251-bib-0021] Montero‐Odasso, M. , M. Speechley , H. Chertkow , et al. 2019. “Donepezil for Gait and Falls in Mild Cognitive Impairment: A Randomized Controlled Trial.” European Journal of Neurology 26, no. 4: 651–659. 30565793 10.1111/ene.13872

[brb371251-bib-0022] Mori, E. , M. Ikeda , E. Iseki , et al. 2024. “Efficacy and Safety of Donepezil in Patients With Dementia With Lewy Bodies: Results From a 12‐Week Multicentre, Randomised, Double‐Blind, and Placebo‐Controlled Phase IV Study.” Psychogeriatrics 24, no. 3: 542–554. 38439118 10.1111/psyg.13091 PMC11578010

[brb371251-bib-0023] Mori, E. , M. Ikeda , and M. Ohdake . 2024. “Donepezil for Dementia With Lewy Bodies: Meta‐Analysis of Multicentre, Randomised, Double‐Blind, Placebo‐Controlled Phase II, III, and IV Studies.” Psychogeriatrics 24, no. 3: 589–596. 38439217 10.1111/psyg.13101 PMC11578031

[brb371251-bib-0024] Pachón‐Angona, I. , B. Refouvelet , R. Andrýs , et al. 2019. “Donepezil + Chromone + Melatonin Hybrids as Promising Agents for Alzheimer's Disease Therapy.” Journal of Enzyme Inhibition and Medicinal Chemistry 34, no. 1: 479–489. 30712420 10.1080/14756366.2018.1545766 PMC6366423

[brb371251-bib-0025] Padovani, A. , S. Falato , and V. Pegoraro . 2023. “Extemporaneous Combination of Donepezil and Memantine to Treat Dementia in Alzheimer Disease: Evidence From Italian Real‐World Data.” Current Medical Research and Opinion 39, no. 4: 567–577. 36803101 10.1080/03007995.2023.2182530

[brb371251-bib-0026] Pérez Palmer, N. , B. Trejo Ortega , and P. Joshi . 2022. “Cognitive Impairment in Older Adults: Epidemiology, Diagnosis, and Treatment.” Psychiatric Clinics of North America 45, no. 4: 639–661. 36396270 10.1016/j.psc.2022.07.010

[brb371251-bib-0027] Petersen, R. C. , R. G. Thomas , M. Grundman , et al. 2005. “Vitamin E and Donepezil for the Treatment of Mild Cognitive Impairment.” New England Journal of Medicine 352, no. 23: 2379–2388. 15829527 10.1056/NEJMoa050151

[brb371251-bib-0028] Raina, P. , P. Santaguida , A. Ismaila , et al. 2008. “Effectiveness of Cholinesterase Inhibitors and Memantine for Treating Dementia: Evidence Review for a Clinical Practice Guideline.” Annals of Internal Medicine 148, no. 5: 379–397. 18316756 10.7326/0003-4819-148-5-200803040-00009

[brb371251-bib-0029] Russ, T. C. , and J. R. Morling . 2012. “Cholinesterase Inhibitors for Mild Cognitive Impairment.” Cochrane Database of Systematic Reviews 2012, no. 9: CD009132. 22972133 10.1002/14651858.CD009132.pub2 PMC6464825

[brb371251-bib-0030] Schuff, N. , N. Woerner , L. Boreta , et al. 2009. “MRI of Hippocampal Volume Loss in Early Alzheimer's Disease in Relation to ApoE Genotype and Biomarkers.” Brain 132, no. 4: 1067–1077. 19251758 10.1093/brain/awp007 PMC2668943

[brb371251-bib-0031] Small, G. W. , L. M. Ercoli , D. H. Silverman , et al. 2000. “Cerebral Metabolic and Cognitive Decline in Persons at Genetic Risk for Alzheimer's Disease.” Proceedings of the National Academy of Sciences of the United States of America 97, no. 11: 6037–6042. 10811879 10.1073/pnas.090106797 PMC18554

[brb371251-bib-0032] Taha, D. E. , A. M. Kabel , A. I. Yassin , and A. A. Abdin . 2025. “The Potential Mitigating Effect of Febuxostat Alone or With Donepezil on Scopolamine‐Induced Alzheimer's Disease in Rats: The Role of TXNIP/NLRP3 Inflammasome Signaling Pathway.” European Journal of Pharmacology 1002: 177875. 40562196 10.1016/j.ejphar.2025.177875

[brb371251-bib-0033] Tang, J. Q. , Q. H. Shen , Y. Y. Han , et al. 2023. “Analysis of Research Status and Trends on Marine Benthic Dinoflagellate Toxins: A Bibliometric Study Based on Web of Science Database and VOSviewer.” Environmental Research 238, no. 2: 117179. 37748671 10.1016/j.envres.2023.117179

[brb371251-bib-0034] van Dyck, C. H. , C. J. Swanson , P. Aisen , et al. 2023. “Lecanemab in Early Alzheimer's Disease.” New England Journal of Medicine 388, no. 1: 9–21. 36449413 10.1056/NEJMoa2212948

[brb371251-bib-0035] Wang, J. Y. , J. Y. Qin , J. Y. Ye , et al. 2024. “The Therapeutic Effects of Noninvasive Brain Stimulation Combined With Cognitive Training in Elders With Alzheimer's Disease or Amnesic Mild Cognitive Impairment.” Journal of Prevention of Alzheimer's Disease 11, no. 1: 222–229. 10.14283/jpad.2024.1 38230735

[brb371251-bib-0036] Wang, W. , M. Liu , Z. Wang , et al. 2025. “Global Research Trends of Peripheral Nerve Surgery: A Bibliometric and Visualized Analysis.” Neurosurgical Review 48, no. 1: 429. 40394396 10.1007/s10143-025-03583-1

[brb371251-bib-0037] Wu, Y. T. , A. S. Beiser , M. M. B. Breteler , et al. 2017. “The Changing Prevalence and Incidence of Dementia Over Time—Current Evidence.” Nature Reviews Neurology 13, no. 6: 327–339. 28497805 10.1038/nrneurol.2017.63

[brb371251-bib-0038] Xiao, L. , J. Tang , H. Tan , et al. 2024. “Efficacy and Safety of *Ginkgo biloba* Extract Combined With Donepezil Hydrochloride in the Treatment of Chinese Patients With Vascular Dementia: A Systematic Review Meta‐Analysis.” Frontiers in Pharmacology 15: 1374482. 39021830 10.3389/fphar.2024.1374482 PMC11251972

[brb371251-bib-0039] Yang, S. , X. Lin , H. Wei , J. Peng , P. Shang , and S. Sun . 2026. “Global Research Trends and Current Status of Xenotransplantation: A Bibliometric Analysis.” Xenotransplantation 33, no. 1: e70104. 41491651 10.1111/xen.70104

[brb371251-bib-0040] Yi, L. , W. Wang , Y. Chen , et al. 2025. “A Bibliometric Analysis of Global Research Trends in Autophagy and Glioblastomas.” Naunyn‐Schmiedeberg's Archives of Pharmacology 00, ahead of print, September 17. 10.1007/s00210-025-04578-x 40960516

[brb371251-bib-0041] Youn, D. H. , Y. Lee , S. W. Han , et al. 2024. “Therapeutic Effect of Donepezil on Neuroinflammation and Cognitive Impairment After Moderate Traumatic Brain Injury.” Life 14, no. 7: 839. 39063593 10.3390/life14070839 PMC11278464

