# Supplementary material for: Donepezil Research in Cognitive Impairment: A Bibliometric and Scientometric Analysis of Global Trends and Pharmacological Perspectives
Source: Brain Behav. 2026 Feb 10;16(2):e71251. doi: 10.1002/brb3.71251 (PMC12891979; doi:10.1002/brb3.71251)
Supplement: Supplementary file 1 — The top 10 most co‐cited references. [file BRB3-16-e71251-s001.docx]

Supplementary File 1: The top 10 most co-cited references.

| **Rank** | **First author** | **Year** | **Journal** | **Title** | **Citations** |
| --- | --- | --- | --- | --- | --- |
| 1 | Giovanni B. Frisoni | 2010 | Nature Reviews Neurology | The clinical use of structural MRI in Alzheimer disease | 1476 |
| 2 | Ronald C. Petersen | 2005 | The New England Journal of Medicine | Vitamin E and Donepezil for the Treatment of Mild Cognitive Impairment | 1382 |
| 3 | Zoe Arvanitakis | 2019 | JAMA | Diagnosis and Management of Dementia: Review | 855 |
| 4 | Michael Grundman | 2004 | JAMA Neurology | Mild Cognitive Impairment Can Be Distinguished From Alzheimer Disease and Normal Aging for Clinical Trials | 762 |
| 5 | Klaus Seppi | 2011 | Movement Disorders | The Movement Disorder Society Evidence-Based Medicine Review Update: Treatments for the non-motor symptoms of Parkinson's disease | 630 |
| 6 | Gary W. Small | 2000 | PNAS | Cerebral metabolic and cognitive decline in persons at genetic risk for Alzheimer's disease | 617 |
| 7 | Parminder Raina | 2008 | Annals of Internal Medicine | Effectiveness of Cholinesterase Inhibitors and Memantine for Treating Dementia: Evidence Review for a Clinical Practice Guideline | 587 |
| 8 | Christopher M. Callahan | 2006 | JAMA | Effectiveness of Collaborative Care for Older Adults With Alzheimer Disease in Primary Care: A Randomized Controlled Trial | 587 |
| 9 | W. R. Brown | 2011 | Neuropathology and Applied Neurobiology | Review: Cerebral microvascular pathology in ageing and neurodegeneration | 582 |
| 10 | N. Schuff | 2009 | Brain | MRI of hippocampal volume loss in early Alzheimer's disease in relation to ApoE genotype and biomarkers | 497 |
